# An Uncommon Feature of Chronic Granulomatous Disease in a Neonate

**DOI:** 10.1155/2016/5943783

**Published:** 2016-10-31

**Authors:** Razieh Afrough, Sayyed Shahabeddin Mohseni, Setareh Sagheb

**Affiliations:** ^1^Department of Pediatrics, Tehran University of Medical Sciences, Tehran, Iran; ^2^Department of Dermatology, Tehran Medical Sciences Branch, Islamic Azad University, Tehran, Iran

## Abstract

Chronic Granulomatous Disease (CGD) represents recurrent life-threatening bacterial and fungal infections and granuloma formation with a high mortality rate. CGD's sign and symptoms usually appear in infancy and children before the age of five; therefore, its presentation in neonatal period with some uncommon features may be easily overlooked. Here we describe a case of CGD in a 24-day-old boy, presenting with a diffuse purulent vesiculopustular rash and multiple osteomyelitis.

## 1. Introduction

Chronic Granulomatous Disease (CGD) is a rare inherited disease of phagocytic system that leads to recurrent and severe bacterial and fungal infections with a high mortality rate [1, 2]. CGD is characterized by granuloma and abscess formation in the skin, liver, lungs, spleen, and lymph nodes. These granuloma and abscess are caused by the inability of macrophages to kill ingested organisms [2, 3]. Infants with CGD encounter life-threatening infections, so prompt diagnosis and treatment with broad spectrum antibiotics are crucial [4, 5]. CGD's sign and symptoms usually appear in infancy and children before the age of five [3]; therefore, its presentation in neonatal period with some uncommon features may be easily overlooked. We report a 24-day-old boy with an uncommon presentation of CGD.

## 2. Case Presentation

A 24-day-old boy was referred to our hospital with vesiculopustular rash in the periorbita, genitalia, foot, and sacroiliac regions. The patient was born to a 26-year-old primigravida woman after a full term gestation without any complications during pregnancy. His father and mother were cousins. His birth weight was 2700 gr. He was admitted to NICU due to respiratory distress and was discharged after 4 days with a healthy condition. Ten days after his birth, he developed a vesiculopustular rash progressively in periorbita, genitalia, foot, and sacroiliac regions.

Fourteen days later, he was referred to our hospital and was admitted for further evaluation and treatment. There was no complaint of poor feeding or fever. In physical examination, his weight was 2950 gr. He was not ill or toxic. Neonatal reflexes were normal.

**Figure 1:**
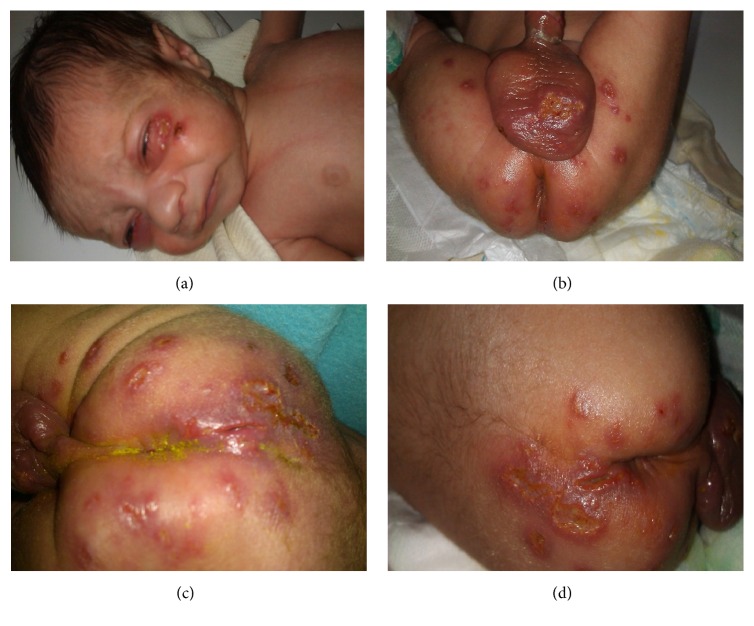
Vesiculopustular lesions ((a)–(d)).

Asymmetric vesiculopustular lesions partially ruptured with erosions and crusted ulcers were seen. They were found in the left periorbital region, scrotum, penis, and sacroiliac region and on the medial malleolus of the left ankle, with some necrosis having extension into the dorsal surface of the foot (Figure 1).

**Figure 2:**
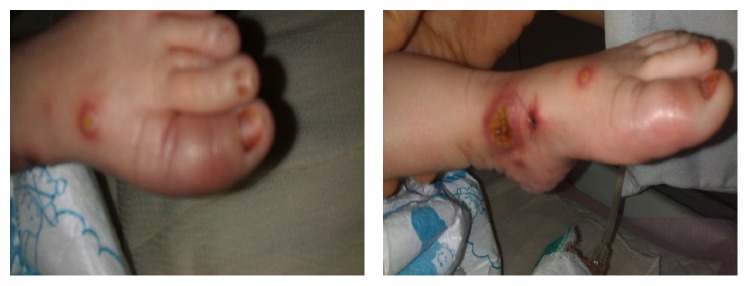
Dactylitis in the left foot.

We also found conjunctivitis with purulent discharge and dactylitis in the left foot. Examinations of other organs were normal (Figure 2).

Routine laboratory tests, smear, and culture from lesions and lumbar puncture were performed (Tables 1 and 2). Chest X-ray was normal at the time of admission, so lung CT scan was not performed.

Gram positive cocci were seen in direct smear from skin lesions, and culture was also positive for* Staphylococcus aureus*. Tzanck smear was negative for the Herpes Simplex Virus (HSV). Samples were sent to determine the specific mutation, but the results are not available yet.

We started our treatment with a combination of broad spectrum antibiotics (meropenem and vancomycin) and local treatment with saline irrigation and sterile dressing and then modified it to vancomycin and amikacin when culture results were available.

**Figure 3:**
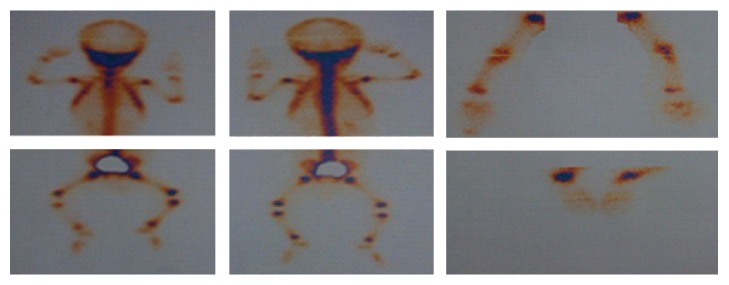
Osteomyelitis of the left ankle, right elbow, and right wrist.

According to the severity and extension of the lesions, a consult with a dermatologist and an immunologist was requested. Skin biopsy showed necrotizing granulomatous tissue reaction, with infectious etiology. Nitroblue tetrazolium (NBT) and Dihydrorhodamine (DHR) tests were performed for confirming diagnosis. Osteomyelitis of the left ankle, right elbow, and right wrist was seen in Tc99m whole body scan (Figure 3).

BCGiosis or tuberculosis was ruled out by biopsy of phalanx.

**Figure 4:**
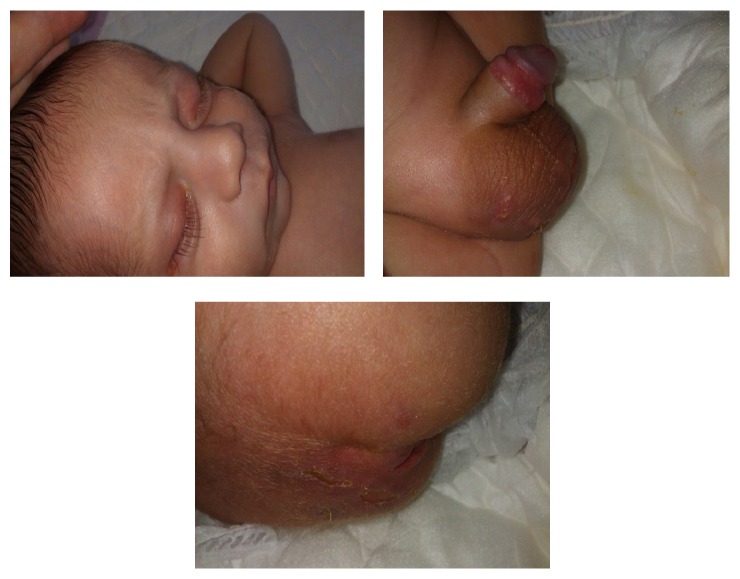
lesions after treatment.

After a few days of treatment, lesions were significantly improved. Treatment with intravenous antibiotics continued for six weeks, and then he was discharged with antibiotic (trimethoprim-sulfamethoxazole) and antifungal prophylaxis (Figure 4).

## 3. Discussion

Chronic Granulomatous Disease (CGD) is an inherited rare disorder of the immune system and represents with recurrent infections and granuloma formation at different sites [5, 6]. Pneumonia, liver abscess, lymphadenitis, osteomyelitis, and skin (cellulitis or abscesses) are the most important clinical manifestations [4–7].

We have encountered an infant with multiple diffuse vesiculopustular lesions with multiple osteomyelitis but there was no evidence of pneumonia and lymphadenitis.* Staphylococcus aureus*, gram negative* Enterobacteriaceae*, and* Aspergillus* species are the most common pathogens [5, 6]. In our patient, Gram positive cocci were seen in direct smear, and culture was positive for* Staphylococcus aureus*. We started our treatment with broad spectrum antibiotics and then modified them to vancomycin and amikacin based on culture results. Diagnosis of CGD is based on the DHR test. This test evaluates neutrophil superoxide production via NADPH oxidase complex [4]. Due to diffused and delayed heeling lesions, the NBT and DHR tests were used as diagnostic tests for CGD. As infants with CGD encounter life-threatening infections, early diagnosis and prompt treatment with antibiotics are crucial during acute infections. Antibacterial and antifungal prophylaxes are considered for reducing infections in CGD [5, 6]. Immunotherapy with interferon-*γ* is sometimes taken and hematopoietic stem cell transplant is also considered in severe forms [8, 9]. In our patient, treatment with intravenous antibiotics continued for 6 weeks and then he was discharged with antibiotic and antifungal prophylaxis.

Previous studies presented multifocal abscess [10] and invasive pulmonary aspergillosis [11] as clinical manifestation of CGD during neonatal periods.

In our case, multiple diffuse vesiculopustular lesions with multiple osteomyelitis were considered as a clinical presentation of CGD.

Despite the rare incidence of CGD during neonatal period, it should be considered in the differential diagnosis of a newborn with clinical features of skin cellulitis or abscesses and multiple osteomyelitis in the absence of appropriate response to treatment with antibiotics.

## Figures and Tables

**Table 1:** Results of the routine laboratory tests.

Parameter	Before treatment	After treatment	Units
WBC	15.2	11.3	K/*μ*L
Neut	55	41	%
Lymph	31.6	32.8	%
Mono	11.9	21.9	%
Eos	1.6	4	%
RBC	4.23	3.75	M/*μ*L
Hgb	13.3	11.5	g/dL
Platelet	112	582	K/*μ*L
CRP	56.2	22	mg/L

**Table 2:** Results of the lumbar puncture.

Parameter	Value
Protein	45 mg/dL
Glucose	57 mg/dL
WBC	1/*μ*L
RBC	700/*μ*L
Smear	Negative
Culture	Negative
PCR for HSV	Negative
